# Profiling the Dead: Generating Microsatellite Data from Fossil Bones of Extinct Megafauna—Protocols, Problems, and Prospects

**DOI:** 10.1371/journal.pone.0016670

**Published:** 2011-01-31

**Authors:** Morten E. Allentoft, Charlotte Oskam, Jayne Houston, Marie L. Hale, M. Thomas P Gilbert, Morten Rasmussen, Peter Spencer, Christopher Jacomb, Eske Willerslev, Richard N. Holdaway, Michael Bunce

**Affiliations:** 1 Ancient DNA Laboratory, School of Biological Sciences and Biotechnology, Murdoch University, Perth, Western Australia, Australia; 2 School of Biological Sciences, University of Canterbury, Christchurch, New Zealand; 3 Centre for GeoGenetics, Natural History Museum of Denmark, Copenhagen, Denmark; 4 Wildlife Identification Laboratory, School of Biological Sciences and Biotechnology, Murdoch University, Perth, Western Australia, Australia; 5 Southern Pacific Archaeological Research, Department of Anthropology, University of Otago, Dunedin, New Zealand; 6 Palaecol Research Ltd, Christchurch, New Zealand; Institut de Biologia Evolutiva - Universitat Pompeu Fabra, Spain

## Abstract

We present the first set of microsatellite markers developed exclusively for an extinct taxon. Microsatellite data have been analysed in thousands of genetic studies on extant species but the technology can be problematic when applied to low copy number (LCN) DNA. It is therefore rarely used on substrates more than a few decades old. Now, with the primers and protocols presented here, microsatellite markers are available to study the extinct New Zealand moa (Aves: Dinornithiformes) and, as with single nucleotide polymorphism (SNP) technology, the markers represent a means by which the field of ancient DNA can (preservation allowing) move on from its reliance on mitochondrial DNA. Candidate markers were identified using high throughput sequencing technology (GS-FLX) on DNA extracted from fossil moa bone and eggshell. From the ‘shotgun’ reads, >60 primer pairs were designed and tested on DNA from bones of the South Island giant moa (*Dinornis robustus*). Six polymorphic loci were characterised and used to assess measures of genetic diversity. Because of low template numbers, typical of ancient DNA, allelic dropout was observed in 36–70% of the PCR reactions at each microsatellite marker. However, a comprehensive survey of allelic dropout, combined with supporting quantitative PCR data, allowed us to establish a set of criteria that maximised data fidelity. Finally, we demonstrated the viability of the primers and the protocols, by compiling a full *Dinornis* microsatellite dataset representing fossils of c. 600–5000 years of age. A multi-locus genotype was obtained from 74 individuals (84% success rate), and the data showed no signs of being compromised by allelic dropout. The methodology presented here provides a framework by which to generate and evaluate microsatellite data from samples of much greater antiquity than attempted before, and opens new opportunities for ancient DNA research.

## Introduction

The discovery and use of polymorphic microsatellite loci (also known as STRs or short tandem repeats) have had significant effects in many areas of genetic research. For the past two decades they have been the markers of choice in a wide range of forensic profiling, population genetics and wildlife-related research. The importance and applicability of these markers are confirmed by observing an excess of 45,000 hits on the word “microsatellite” on the Web of Science database (accessed late-2010). With the development of high-throughput sequencing platforms, such as the GS-FLX (Roche, Branford, CT, USA), microsatellite marker development has recently become fast and efficient [1]–[3] and the advance has also allowed the identification of these loci in highly degraded ancient DNA (aDNA), where traditional enrichment procedures have been unsuccessful [2].

Here, we present the first set of microsatellite markers to be developed directly from aDNA templates with the specific aim of studying an extinct taxon: the New Zealand moa (Aves: Dinornithiformes). The moa lineage included nine species [4] of large herbivorous birds (15–250 kg). They occupied most areas within New Zealand until their sudden extinction shortly after Polynesians colonised the landmass in the late 13^th^ century [5]. However, bones, coprolites, feathers and eggshell of moa are discovered regularly and some natural Holocene sites have yielded fossils in high concentrations e.g., [6], [7], [8] with very good DNA preservation [9]–[12]. These offer rare, if not unique opportunities to study an extinct megafauna at the population level. As demonstrated recently [10], analyses of well-preserved aDNA from hundreds of moa individuals found in close spatial and temporal proximity can uncover patterns in the biology and local population dynamics almost equivalent to research on living (extant) populations. However, to fully extract and explore the information within this paleobiological context, a set of high resolution genetic tools is necessary. It has been shown that nuclear DNA (nuDNA) from moa, such as gender-specific sexing markers [10], [13], [14], and a single previously characterised microsatellite [2], can be amplified with a considerable success rate. Because microsatellites have a high mutation rate and bi-parental inheritance, they can offer more detailed population genetic insight than maternally inherited mitochondrial DNA (mtDNA). A fully functional set of microsatellite markers would therefore allow research into moa biology, population structure, and the extinction process in much greater detail than has been attempted before with aDNA for any taxon.

The procedure for identifying microsatellites from aDNA has already been established [2], so the two major challenges in the present study were: 1) to identify and characterise a sufficient number of useable and polymorphic loci to yield informative data and 2) to investigate whether data quality was compromised by having been generated from LCN aDNA templates. Given good DNA preservation, the first issue is essentially a matter of time and resources to identify, design, and screen enough primers. The second issue is, however, potentially more problematic. Ancient DNA research generally targets mtDNA because the relative copy number is much higher than their nuclear counterparts. Aiming at nuclear markers such as microsatellites is far more challenging, and requires stringent methodology to ensure data fidelity.

The main concern, apart from complete PCR failure, is ‘allelic dropout’. This dropout effect can be observed when amplifying LCN DNA, because the stochastic nature of the earliest cycles in the PCR reaction can cause one of the two (in diploids) alleles to ‘swamp’ the PCR, completely suppressing amplification of the other allele e.g., [15]. The outcome is a genetic profile resembling that of a homozygote regardless of the true genotype of the sample. Allelic dropout is well known in PCRs with LCN DNA [16]–[19] and has been observed and assessed in DNA extracts from a variety of substrates, including faeces [20]–[22], hair [22], [23], feather [24], tooth [25], and fingerprints [26]. The magnitude of the allelic dropout can vary significantly between samples, between different primers, and also between alleles within the same microsatellite marker, so a case-by-case assessment is necessary to establish the extent of the problem and the required protocols. Most important in this context, is to determine how many independent PCR repeats are necessary before the genotype of an apparent homozygous individual can be reliably assigned. This is known as the ‘multiple tubes’ approach, which was first applied and investigated systematically in Navidi et al. [16] and Taberlet et al. [18].

For our ancient moa DNA, it was apparent very quickly that dropout was affecting the allele scoring. Therefore, following the successful identification of suitable microsatellites, DNA extracts of the South Island giant moa (*Dinornis robustus*; *n* = 88) from fossil locations in North Canterbury, were screened. Doing so allowed us to characterise in detail the allelic dropout in each marker, and to establish a set of criteria to minimise artefacts and maximise data fidelity. To test the viability of the established protocols, a full multi-locus *Dinornis* dataset was assembled and examined thoroughly for signs of allelic dropout, such as a deficit of heterozygous individuals in comparison to the expected Hardy-Weinberg proportions. Concurrently, results of a comprehensive quantitative PCR (qPCR) experiment on the extracts were used to investigate a link between DNA preservation and dropout and also between DNA preservation and individual observed homozygosity.

In addition to making the first aDNA microsatellite markers available to research, we envisage that the methodologies and considerations outlined here will greatly assist in generating reliable microsatellite DNA profiles from degraded templates. This will be of use in the field of aDNA but also in related disciplines including molecular forensics and wildlife genetics.

## Materials and Methods

### Sampling and DNA extraction

**Figure 1:**
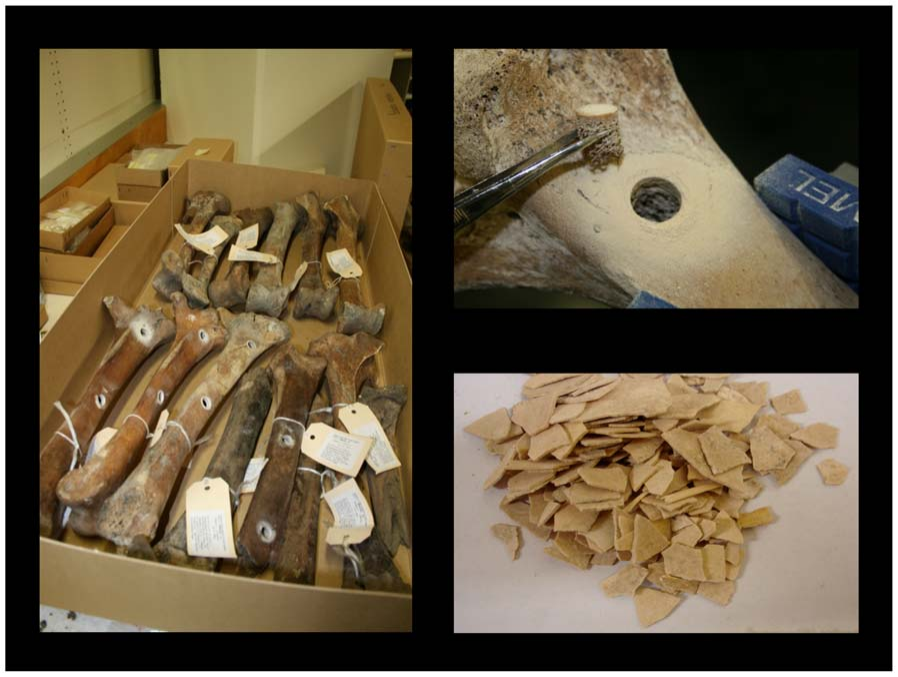
Moa fossils. Moa long bones (left tibiotarsa) and moa eggshell were sampled for aDNA extraction. Photos: MEA and CO.

A constant amount of bone powder (200 mg) from each sample (Figure 1) was digested with proteinase K. DNA was extracted using Centricon 30.000 MWCO ultra-filtration columns (Millipore, Billerica, MA, USA) followed by a silica based purification method (Qiagen, Valencia, CA, USA) to effectively remove PCR inhibitors [27]. DNA was always eluted in a volume of 50 µl to ensure all extracts were comparable in the qPCR assays. Detailed descriptions of the samples, bone sampling protocols, DNA extractions, PCR conditions and sequencing for species identification are given in Allentoft et al. [2], [10]. The moa eggshell DNA used for the GS-FLX run was extracted according to protocols in Oskam et al. [9]. In accordance with aDNA guidelines, all DNA extractions and PCRs reported in this study were performed in a separate and dedicated aDNA laboratory at Murdoch University (Perth, Australia).

### High throughput sequencing

The Moa_MS2 microsatellite was described earlier in a ‘proof of concept’ study based on data from a Roche GS-FLX run [2]. To identify additional polymorphic microsatellites, further GS-FLX data were generated from a total of 10 moa DNA extracts (Table 1). Six of these extracts were prepared as conventional FLX shotgun libraries and each was sequenced on 1/8^th^ of a PicoTiter plate (Roche) using LR70 chemistry. The FLX process followed the manufacturer's guidelines with the following exceptions: 1) As the aDNA was likely fragmented *a priori* from post mortem damage, initial fragmentation was omitted. 2) Library release from beads before quantification used the heat-release protocol advocated by Maricic and Pääbo [28]. 3) Before emPCR, the DNA quantity in each library was determined using qPCR [29].

**Table 1:** GS-FLX results.

Taxon	Extract #	Substrate	Run #	Plate	Reads	Av. length	STRs	%
*D. robustus*	MB146	eggshell	1	1/8	16572	133.4	116	0.70
*D. robustus*	MB147	eggshell	1	1/8	21090	122.4	33	0.16
*D. robustus*	MB149	eggshell	1	1/8	19956	119.6	119	0.60
*D. robustus*	MB550	bone	1	1/8	27829	113.1	72	0.26
*D. robustus*	PIBH16A	bone	1	1/8	40007	104.4	25	0.06
*P. elephantopus*	MB551	bone	1	1/8	40447	107.6	9	0.02
*D. robustus*	DBHS	eggshell	2	MID	85476	123.7	37	0.04
*D. robustus*	MB572	eggshell	2	MID	125254	120.4	566	0.45
*P. mappini*	MB595	eggshell	2	MID	20934	85.5	14	0.07
*A. didiformis*	MB718	eggshell	2	MID	206208	119.1	818	0.40
Total from eggshell in run #1			1		57618	124.6	268	0.47
Total from bone in run #1			1		108283	107.8	106	0.10
Total from eggshell in run #2			2		437872	118.8	1435	0.33
Overall					603773	115.4	1809	0.30

Summary of the output from two GS-FLX runs. Ten moa DNA extracts were used, representing two types of substrate and four moa species. *D*, *Dinornis*; *P*, *Pachyornis*; *A*, *Anomalopteryx*. The first run (#1) was conducted using traditional 454-libraries and each of the six moa extracts occupied 1/8 of a PicoTiter plate. Run #2 was performed with pooled MID-tagged libraries added to a full plate. *Reads* and *Average length* describe the total number of filtered sequences and their average length. *STRs* is number of identified microsatellites (≥6 repeat units) and *Percentage* (%) denotes the proportion of reads with microsatellites.

The remaining four extracts were prepared as MID-tagged libraries with the same (as above) modifications to the manufacturer's guidelines. Following emPCR, approximately equal quantities of enriched beads from each library were pooled into a single library, and sequenced on a full PicoTiter plate using LR70 chemistry. Prior to data analysis, the sequence reads from this dataset were sorted by MID-tag into the original four libraries.

### Microsatellite primer design and optimisation

The sequences obtained were screened for repetitive nucleotide regions using MSATCOMMANDER [30]. The candidate sequences were inspected individually, to select the sequences showing a large number of repeat units, as well as sufficient flanking region and base composition, suitable for primer design. Microsatellite primers were then designed using the web-based program PRIMER3PLUS [31], with settings ensuring short PCR products of ∼100 bp, to maximize their applicability to degraded LCN aDNA. Each primer pair was then tested and optimised on DNA extracts from South Island giant moa (*Dinornis robustus*), using the 50-cycle microsatellite PCR protocol presented in Allentoft et al. [2], but with variable annealing temperatures optimised for each primer set (Table 2). Given the relative difficulty in obtaining well-preserved samples and extracting DNA from fossil bone, all PCR reactions were done in relatively small volumes (a total of 12.5 µl per reaction rather than 25 µl as in [2]), using just 1 µl of the undiluted DNA extract per reaction. PCR products were screened with electrophoresis on 2% agarose gels. If bands were visualised in the expected size-range, primers were re-ordered with a 5′ fluorescent 6-FAM dye (Integrated DNA Technologies, Coralville, IA, USA) and the PCRs were repeated with these. DNA fragments were separated on an ABI 3730 genetic analyser and sized with Genescan LIZ500 size standard (Applied Biosystems, Foster City, CA, USA). Negative and positive controls were always included. The alleles were scored using GENEMARKER version 1.5 (Soft Genetics, State College, PA, USA).

**Table 2:** Characteristics of the six microsatellite primer pairs.

Locus	Source taxon	Identified repeat	Ann. temp.	Forward 5′→ 3′	Reverse 5′→ 3′
Moa_MS2	*P. elephantopus*	(AC)_12_	57°C	GAGCACCAATACAACTTCATGG	GACTGTTATTCTATTCCAGTATATGTTTG
Moa_MA1	*D. robustus*	A_7_(CA)_9_	55°C	CATATAGGCACAAGGAGAGC	CAGGGGAGGATGGTATCTGT
Moa_MA21	*A. didiformis*	(GT)_9_	57°C	CGTGTCTCGGATGCATAGAT	GTTATCTGTGCGCCTTGC
Moa_MA38	*A. didiformis*	(AC)_8_	58°C	GCTTGTTCCCTCCATCACAT	CAGCACTGTGCAGCACTTTC
Moa_MA44	*A. didiformis*	(AC)_8_	58°C	AGGATTAGATCCCAGGAAGC	CTCTCAGCCTGTGGACTTTG
Moa_MA46	*A. didiformis*	(GAG)_9_	66°C	GGCTGTCCGCCACTCAAG	GAGAAGGGCTCGGTCCTC

Characteristics of the six primer pairs, including the moa species in which the microsatellites were identified, repeat units as observed in the original GS-FLX clones, the annealing temperatures in the 50-cycle PCR reaction from Allentoft et al. [2], and the primer sequences.

### Allelic dropout, summary statistics and HW-tests

In a large pilot study, an allele-call database was established based on more than 1500 successful PCR reactions across the six markers, using 88 available *Dinornis robustus* DNA extracts (representing the same number of individuals, as the extracts were derived from 88 left tibiotarsi bone elements). Allelic dropout for each marker was calculated as the proportion of PCRs in known heterozygotes that amplified only one of the two alleles. A heterozygote was simply determined as showing either a clear bi-allelic profile in one PCR, or two different mono-allelic profiles in different PCRs. Hence, a heterozygote individual showing AA, BB, BB, and AB in four different successful PCRs would exhibit a dropout fraction of 75%, whereas a heterozygote showing AB, AB in two different PCRs would have 0% dropout.

Each individual was successfully genotyped an average of 2.9 times for each of the six markers, although homozygotes and problematic individuals (showing signs of allelic dropout) were generally repeated more intensively in this pilot study than individuals showing clear bi-allelic profiles. We note that this selectiveness may have led to a slight exaggeration of the estimated dropout fractions compared to data generated from a random PCR-repeat schedule. From a methodological viewpoint this is not a concern as an overestimate of allelic dropout can only lead to more conservative protocols, ensuring higher data fidelity.

Basic descriptive statistics of the final assembled microsatellite data, including measures of genetic diversity (*N*_A_, *N*_E_, *H*_O_, *H*_E_) and fixation indices (*F*_IS_), were calculated using MSTool (Park, 2001) and GenAlEx v. 6.4 [32]. Tests for deviations from Hardy-Weinberg proportions within and across the microsatellite markers, and linkage disequilibria between markers, were performed with the methods implemented in GENEPOP v.4.0.10 [33]. MICRO-CHECKER v. 2.2.3 [34] was used to further assess the quality of the data by looking for evidence of scoring errors resulting from stuttering, null alleles, or drop out of long alleles.

A ‘Probability of Identity’ (*PI*) assessment was carried out as implemented in GenAlEx. The *PI* estimates the probability that two unrelated individuals in the dataset, will by chance have the same multilocus genotype. This information is relevant when evaluating the resolution power of the selected combination of markers, and hence their applicability and informativeness in genetic analyses [35], [36].

### Quantitative real-time PCR

A large proportion of our available moa DNA extracts had already been screened (with the moa specific 262F/441R mtDNA Control Region primers, ∼250 bp) in a quantitative real-time PCR (qPCR) assay. The assay was based upon SYBR detection chemistry on a Bio-Rad My-IQ thermocycler, as described previously [2], [10]. The C_T_ values were recorded as proxies for the relative DNA preservation in each extract. A high C_T_-value reflects a late amplification in the monitored PCR reaction because of a low number of suitable template molecules (in the absence of PCR inhibitors). The accuracy and applicability of our qPCR setup has previously been evaluated, and a clear link between the recorded mtDNA C_T_-values and the ability to amplify nuDNA in these extracts has been demonstrated [2], [9], [10]. To ensure consistency, aDNA extracts were always run with qPCR immediately following the DNA isolation. As outlined further below, the qPCR assessment provided a valuable resource when validating the fidelity of the generated microsatellite data.

## Results

### GS-FLX sequencing

Results for the two GS-FLX runs representing ten aDNA extracts, two types of substrate, and ten moa genera, are shown in Table 1. In total, 603,773 sequences were generated and 1809 relevant repetitive regions (di-, tri-, and tetra-nucleotides with six or more repeat units) were detected among those, corresponding to 0.3% of the sequences. Although the number of sequences in the two different GS-FLX runs cannot be directly compared because of the different sequencing volumes performed (i.e. separated lanes contra MID-tagging), the data suggest that the eggshell extracts provided longer average read lengths and also a higher fraction of potential STRs. Notably, within the first run (Table 1), the three eggshell extracts combined, produced an average of 0.47% STRs and an average read length of 124.6 bp, whereas the same parameters for bone were 0.1% and 107.8 bp, indicating that substrate selection is an important consideration when shotgun sequencing (see Discussion).

### Primer design and initial testing

Although 1809 repetitive regions were detected, only a small fraction of them proved suitable as potential microsatellite markers. The reasons for the large rejection of candidate sequences are commonly observed and include either unsuitable base composition (for example, too high/low GC content), or more often, because insufficient flanking region was available to attach the primers. We designed and tested 61 primers from the GS-FLX data but also tried primers developed for other ratites [37]–[42]. None of these previously developed ratite primers proved suitable. In addition, several generic bird microsatellite markers [43] were redesigned (shortened) for aDNA purposes and tested. One of these amplified the right product in *Dinornis robustus* but proved monomorphic (Table S1). A total of 89 primer pairs was evaluated in the process, across a range of PCR conditions. Ultimately, only five (5.6%) proved suitable (plus the already described Moa_MS2) as genetic markers (Table 2), and met the authentication criteria established in Allentoft et al. [2]. The remainder either failed to amplify anything or amplified non-specifically (86.5%), or amplified the correct target but were not polymorphic (7.9%). However, it should be noted that all the DNA extracts were from individuals excavated from the same area (North Canterbury, South Island); some of these loci classified as monomorphic or dimorphic (listed in Table S1) could be more variable in *Dinornis* from other parts of New Zealand. The Moa_MS2 microsatellite was described previously, but had not been tested on a large sample nor assessed for allelic dropout. It was therefore included in this study to provide a full characterisation of the entire moa microsatellite marker set. The original GS-FLX reads, from which the five new primer sets were designed, have been submitted to GenBank (HQ823574 - HQ823578) and the sequences are shown in Figure S1.

In an attempt to determine the chromosomal position of each of the six markers, the flanking regions were queried against the two presently mapped bird genomes, chicken (*Gallus gallus*) and zebra finch (*Taeniopygia guttata*), using BLAST (www.blast.ncbi.nlm.nih.gov/Blast.cgi). However, because of the short flanking regions and probably also because of the distant relationships of these taxa with moa (>100 million years), the queries were largely unsuccessful. One potential match was that the 145 bp long flanking region in Moa_MA44 showed 83% identity with an 83 bp sequence on the zebra finch chromosome 1, and 96% identity with a 26 bp sequence on the chicken chromosome 1. This marker probably, therefore, resides on the moa chromosome 1. In the future, a reference ratite genome is likely to facilitate a more reasonable approximation of the mapping of these loci.

Given that the six microsatellite primer pairs worked for *Dinornis robustus*, but were designed from DNA of three different moa genera (Table 2), it was highly probable that the markers could amplify across moa taxa. To confirm this, the primers were tested on a few DNA extracts from three other moa genera (*Euryapteryx*, *Emeus* and *Pachyornis*) and all amplified well (data not shown).

### Allelic dropout

**Figure 2:**
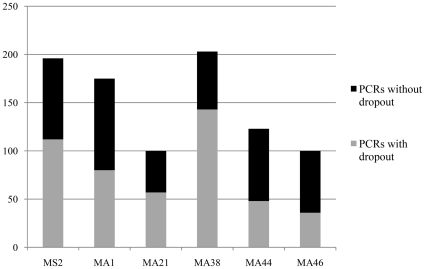
Allelic dropout. Graphical representation of allelic dropout in each marker. Bars show the total number of successful PCR reactions in heterozygote individuals, with the dropout (grey) and no-dropout (black) fractions indicated.

In the pilot study assessing allelic dropout, a total of 897 PCR reactions represented heterozygous individuals, according to the criteria for heterozygosity established above. Overall, 53% of the 897 PCRs showed dropout (displaying only one allele), but the fraction varied substantially between the markers, from 36% in Moa_MA46 to 70% in Moa_MA38 (Table 3, Figure 2). In three of the markers (Moa_MS2, Moa_MA38, Moa_MA46), short allele dominance [15] was evident, whereas in the three other markers, the long, and short alleles dropped out with almost equal frequency (Table 3).

**Table 3:** Assessing allelic dropout.

	MS2	MA1	MA21	MA38	MA44	MA46	Overall
PCRs in heterozygotes	196	175	100	203	123	100	897
PCRs with dropout	112	80	57	143	48	36	476
Observed dropout rate	0.57	0.46	0.57	0.70	0.39	0.36	0.53
PCRs, short allele dropout	39	40	29	44	23	14	189
PCRs, long allele dropout	73	40	28	99	25	22	287
Prob., 2x same allele dropout	0.178	0.104	0.163	0.285	0.076	0.068	
Prob., 3x same allele dropout	0.060	0.024	0.046	0.126	0.015	0.013	
Prob., 4x same allele dropout	0.021	0.005	0.013	0.059	0.003	0.003	
Prob., 5x same allele dropout	0.007	0.001	0.004	0.028	0.001	0.001	
Mean C_T_ in ‘dropouts’	32.46	32.89	32.94	32.51	32.31	33.1	
± SD	±3.08	±3.11	±3.08	±2.81	±2.81	±2.60	
Mean C_T_ in ‘no dropouts’	30.92	31.36	31.44	29.75	33.3	31.82	
± SD	±2.64	±3.04	±2.07	±1.98	±3.18	±2.75	
*t*-tests, *P = *	0.100	0.095	0.090	0.060	0.280	0.110	0.008
	df = 50	df = 53	df = 43	df = 53	df = 43	df = 46	

An assessment of allelic dropout based on 897 PCRs of known heterozygous individuals. The observed dropout rate was calculated for each marker, and the number of reactions showing long allele dropout and short allele dropout are shown. These values were used to estimate the average probability of misidentifying a heterozygote as a homozygote, if applying a multiple PCR repeats approach. Estimated error rates are indicated here for 2–5 PCR repeats. C_T_ mean values (± st.dev.), as a proxy for DNA preservation, were calculated for DNA extracts showing *dropout* and *no dropout* respectively. *P* -values represent the significance of difference in mean values. Overall *P*-value was calculated with Fisher's approach.

Given the observed dropout rates, we calculated the probability that a heterozygote would be misidentified as a homozygote because of dropout. For example; the estimated average probability that the same allele drops out in four consecutive PCRs when using the Moa_MS2 primers, is the probability for the long allele to drop out four consecutive times  =  (73/196)^4^ *plus* the probability that the short allele drops out four consecutive times  =  (39/196)^4^, or 2.1%, as shown in Table 3. In other words, if a 4xPCR-repeat approach was applied for Moa_MS2, a hypothetical 2.1% of the heterozygous individuals were likely to be misidentified as homozygous. Given that expected heterozygosity (*H*_E_) for this locus is 0.794 (79.4% heterozygotes, Table 3), it corresponds to ∼1.7% of the total number of individuals being mis-identified when using a 4x PCR approach. When summed across all six markers, 6.6% (or 4.9 of the 74 individuals) were likely to be incorrectly genotyped as homozygous in one of the six markers. This number has to be considered a theoretical *maximum* value, because it assumes that the estimated number of mis-scoring events was distributed across as many individuals as possible (i.e. a maximum of one error per individual), rather than the more realistic scenario of a few problematic individuals accumulating more errors.

### Establishing the criteria for data fidelity

With the level of allelic dropout assessed for each marker, there was basis for a set of rules for generating reliable moa microsatellite data. The rationale behind the three following criteria is outlined in the Discussion:

**1)** An apparent homozygote was accepted as such only when it showed the same unambiguous mono-allelic profile in at least four independent PCR reactions.

**2)** An apparent heterozygote was accepted as such if: A) it showed an unambiguous bi-allelic profile, and/or, B) it displayed two different unambiguous mono-allelic profiles (representing already well-established alleles) in different PCRs.

**3)** All data from individuals that could not, despite several attempts, be reliably genotyped with at least five of the six microsatellite primers, were removed from the compiled dataset.

Applying these rules, we derived a multilocus microsatellite genotype for 74 of the 88 *Dinornis robustus* extracts – a success rate of 84%.

### Summary statistics, HW-proportions and linkage

All six loci were polymorphic, displaying between five and 17 alleles per locus (Table 4). Expected heterozygosity (*H*_E_) ranged from 0.61–0.84 (Overall 0.72±0.036 SD; Table 4). In general, observed (*H*_O_) and expected heterozygosity proved similar, with only small deviations from Hardy-Weinberg proportions (overall *F_IS_* = 0.04±0.05 SD). When tested, these deviations were not significant except for Moa_MA21, which displayed a small, but significant, heterozygote deficiency (*F_IS_* = 0.109, *P* = 0.04). When Bonferroni-correction was applied to accommodate for possible statistical type I error, the deviation was no longer significant. Further, MICRO-CHECKER [34] found no signs of scoring errors resulting from stuttering, long allele dropout, or null alleles in the data (see Figure S2). Lastly, no signs of linkage disequilibrium were apparent for any of the 15 pair-wise locus combinations (*P*≥0.1).

**Table 4:** Microsatellite summary statistics.

Locus	*n*	Range	*N*_A_	*N*_E_	*H*_O_	*H*_E_	*F_IS_*	*P*
Moa_MS2	72	110–148 bp	16	4.8	0.778	0.794	0.020	0.674
Moa_MA1	74	91–99 bp	7	3.5	0.730	0.715	−0.020	0.897
Moa_MA21	74	93–113 bp	6	3.3	0.622	0.698	0.109	0.040
Moa_MA38	72	84–132 bp	17	6.1	0.792	0.837	0.054	0.331
Moa_MA44	74	75–85 bp	5	2.8	0.595	0.641	0.073	0.257
Moa_MA46	74	66–81 bp	6	2.6	0.608	0.610	0.003	0.238
Overall	74	66–148 bp	9.5	3.8	0.687	0.721	0.040	0.227
± SD			±5.47	±1.37	±0.022	±0.036	±0.048	

Summary statistics for the six loci and 74 *Dinornis robustus* individuals. Number of successfully genotyped individuals (*n*), according to the three established criteria. *Range* is observed allele size range, *N*_A_ is total number of observed alleles, *N*_E_ is effective number of alleles, *H*_O_ and *H*_E_ are observed and expected heterozygosity, fixation index *F_IS_* measures deviations from Hardy-Weinberg proportions, with *P* representing the significance of this deviation. Overall *P* is the Fisher's *P*-value for combined probabilities.

The ‘probability of identity’ assessment yielded a *PI* value of 1.99E-6, reflecting a set of markers with high discrimination power. This means that genetic identity in these data must reflect true relatives and not a random association. The result shows that the markers are informative and confirms their applicability.

### Quantitative PCR and dropout

**Figure 3:**
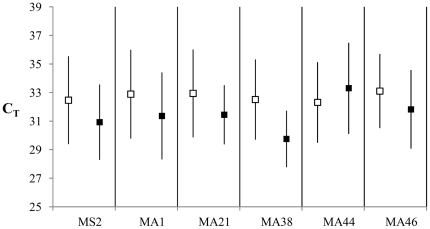
Dropout and C_T_. Graphical representation of differences in average C_T_ values in extracts with allelic dropout (empty boxes) and extracts with no dropout (black boxes). Higher C_T_ values are accompanied by higher chance of dropout in five of the six markers. Exact values are listed in Table 3.

To test for a relationship between DNA preservation and allelic dropout, the mtDNA CR C_T_-values recorded from qPCRs were used as proxies for preservation in the samples. Of the 74 DNA extracts included in the compiled dataset, an accompanying C_T_ value was available from previous studies for 66 [2], [10]. Although a relationship between dropout rate and C_T_ value was expected, the correlation is complicated by individuals with long alleles having a higher chance of showing dropout, regardless of their preservation state. Therefore, the analysis was kept simple, with individuals separated into two groups, showing dropout and no dropout, respectively. The average C_T_ values for the groups were then compared. Although not significant (*t*-tests) the expected pattern of a higher average C_T_ values in dropout-individuals was confirmed in all markers except for Moa_MA44 (Table 3, Figure 3). When *P*-values were combined across loci with Fisher's approach, it was demonstrated with high significance (*P* = 0.008) that individuals prone to allelic dropout were also accompanied by higher average C_T_-values than individuals without observed dropout (Table 3).

**Figure 4:**
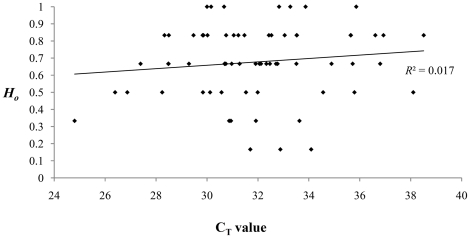
Heterozygosity and C_T_. Evidence that observed individual heterozygosity (*H*_O_) among 66 *Dinornis* individuals in the final dataset is not determined by DNA preservation (represented by C_T_ values). If that was the case, a negative correlation would be expected here.

This confirmation allowed for a direct investigation of the integrity of the full *Dinornis* dataset. If low individual heterozygosity (proportion of heterozygous loci in an individual) was a simple effect of high allelic dropout, a negative correlation would be expected between the observed heterozygosity (*H*_O_) and the recorded C_T_ value. The results presented in Figure 4 suggest that this is not the case (*R* ^2^ = 0.017). There is no negative correlation to suggest that individual observed heterozygosity in the final data has been influenced by DNA preservation.

## Discussion

From this study, we have shown that it is possible to generate a fully functional microsatellite library from LCN aDNA templates. Several studies have amplified microsatellite markers from more recent museum specimens (i.e. decades to a few centuries old), for example to investigate a loss of genetic biodiversity resulting from recent environmental changes e.g., [44], [45]–[47]. Here, we show that reliable microsatellite data can be generated from samples of much greater antiquity, but that care needs to be exercised in compiling the data.

### High-throughput sequencing

This study demonstrates that it takes a considerable effort to retrieve appropriate microsatellite markers from aDNA. From >600,000 GS-FLX sequences, >1800 detected STRs, and 89 tested primer-pairs, a total of six functional microsatellite markers may seem a rather poor return. This difficulty is a consequence of the fragmented nature of aDNA, but it also reflects the capacity of the GS-FLX Standard platform in terms of read lengths. The more recent GS-FLX Titanium chemistry, with longer read lengths, is likely to improve the success rate, and so is the introduction of a successful STR enrichment step before the 454-sequencing [3]. Differing DNA quality probably explains the observed variation in results between extracts (Table 1). In terms of number of reads, average read lengths, and number of detected STRs, the eggshell extracts appear to perform better than the bone extracts, apart from one very poor eggshell extract, with a short average read length (MB 595). This agrees with a recent study demonstrating high DNA quality in ancient eggshells, including eggshell from moa [9]. The better return from the eggshell runs may result from a lower bacterial load in this substrate. The *Anomalopteryx* eggshell (MB 718), in particular, produced many reads, and yielded four of the six microsatellites described here.

Whereas our combined data showed an average of 0.3% of the sequences containing repeats (1809 of 603,773 sequences), values such as 2.4% [48], 1.8% [49], 1.3% [1], and 11.3% [50] have been demonstrated when mining for microsatellites in modern DNA. Although the numbers are not strictly comparable because of slight variations in the STR search-criteria and inter-specific variation in STR frequency, it seems that aDNA produces a considerably smaller fraction of sequences with STRs. In animals, microsatellites are generally found only in the nuclear genome, so this difference perhaps reflects the scarcity of nuclear DNA in the fossils, or that microbial DNA contributes to a high proportion of the reads, or perhaps that microsatellites were simply relatively rare in moa, as has been suggested for other birds [51]. Future work on extinct mammals may clarify this situation.

### Allelic dropout

It is clear from the data that allele scoring was seriously hampered by allelic dropout (Table 3). Indeed, if the common approach with just one PCR per marker was applied, an overall average of 53% of the heterozygotes would likely have been misidentified as homozygotes. Likewise, attempts to multiplex even two of these markers together caused even greater dropout and cannot be advised for templates such as these. As mentioned above, the problem is well described in the literature although the average dropout we observed seems slightly worse than in studies of, for example, faecal DNA: 24% dropout in Morin et al. [22] and 29% dropout in Frantz et al. [21], DNA from 100-year-old teeth: 42% dropout in Arandjelovic et al. [25], shed hair: 31% dropout in Gagneux et al. [52], and human fingerprints: 12% in Balogh et al. [26]. The three criteria, proposed to minimise the effect of dropout on our moa data are discussed below:

**Criterion 1** involved a 4x singleplex PCR repeat method (multiple tubes approach) for each apparent homozygote. According to the average error rates shown in Table 3, this should reduce genotyping errors to affect between 0.3% and 5.9% of the heterozygotes per marker. When summed, a theoretical maximum of 6.6% of all genotyped individuals in the final data would contain an error. This 4x approach was based on balancing the generation of high quality data, against maintaining enough DNA extract to amplify all markers. The evaluation had to factor in the relative difficulty in obtaining sample material for additional extractions, and also account for a high rate of PCR failures, given that retrieving results from four positive PCR reactions could for some extracts easily involve 6–10 PCR setups. Our approach here is less conservative than the often cited 7x PCR repeat suggested by Taberlet et al. [18], which was based on computer simulations of a worst-case scenario with 100% allelic dropout. Rather than automatically assuming a worst-case scenario, we measured the level of dropout directly in each marker and relied on a less stringent multiple tubes approach, more comparable to protocols suggested elsewhere e.g., [17], [21]. It is well known that genotyping errors are present in most microsatellite datasets [53] and the theoretical 6.6% genotyping error rate we report here is not higher than what is expected, or observed, for ‘modern’ data [53]–[55].

**Criterion 2** included the genotyping of heterozygotes, which we accepted as individuals showing two distinct (and already well-established) alleles in any number of PCR reactions (i.e. two different alleles in the same PCR or two different alleles in separate PCRs). Other studies have suggested stricter rules, arguing, for example, that an allele must be recorded at least twice in each individual to be accepted [18], [21]. For a large proportion of our samples, each allele was indeed recorded more than once, but we found no support in the data for applying this as a criterion. The concern in this context is that a contamination event or lack of specificity in the PCR reaction can lead to scoring of false alleles. Both these problems might theoretically arise more frequently in PCRs using aDNA templates. However, the problem appeared so rarely that it should not set a precedent for a rule. Amongst the >1500 successful PCR reactions, we recorded fewer than 10 reactions with alleles that could not be accounted for. *False* peaks (the result of non-specific amplification) appeared regularly though with two of the markers (such as a distinct 115 bp peak with Moa_MA38), but these lacked the characteristic pattern of microsatellite profiles such as stutter peaks, and could hence easily be identified and eliminated. Contamination is potentially a more serious problem and it is possible that an allele observed only once can represent cross-contamination from another sample. However, this also applies to an allele observed twice. If a sample is contaminated, multiple PCRs will not necessarily clarify the situation. Conversely, if cross-contamination happens occasionally during PCR setup, confirmation in multiple repeats should effectively remove its effect. We are, however, confident that there was very little cross-contamination evident in our data. Because of the difficulty in obtaining moa samples, DNA extracts were stored in separate 1.5 ml Eppendorf tubes to minimise evaporation. Each extract tube was opened individually and DNA extract was single-pipetted into each respective PCR tube (not 96-well plates), which involved much less risk of cross-contamination than do the commonly applied multi-pipette PCR setups. Additionally, extractions and PCRs were performed in a dedicated clean lab using aDNA guidelines. These strict measures may explain why we did not observe contamination issues in the *Dinornis* data, either here nor in previous work based on the same DNA extracts [2], [10].

**Criterion 3** involved the removal of problematic individuals from the dataset with the specific aim of reducing the theoretical 6.6% error rate further. Rather than using indirect indications for the potential genotypic reliability of each sample, based, for example, on the qPCR assays, we took a more direct approach, and removed individuals that could not amplify in at least five of the six microsatellites despite several attempts. It is clear that successful PCR amplifications in different loci constitute strong reciprocal support for the results. For example, if a long allele has been identified in one locus, it is a confirmation that sufficient nuclear DNA preservation exists (in that extract) for relatively long alleles in the other loci to be amplified as well. On the other hand, if a given DNA extract can amplify only one or two of the microsatellite loci despite repeated attempts, the argument collapses and all data for that individual should be removed from the compiled dataset.

### Interrogation of the data

Interestingly, a relatively high level of genetic diversity was documented for the *Dinornis robustus* sample (*H*_E_ = 0.72, Table 4). The value is comparable to, or perhaps slightly higher, than heterozygosity estimates of ostrich (*Struthio camelus*) subspecies (average *H*_E_ ranged from 0.30 to 0.71), which appears to be the only other ratite that has been studied with microsatellite analyses [56]. The level of genetic diversity we recorded for *Dinornis*, combined with the discrimination power documented in the ‘Probability of Identity’ analysis, suggest that these six markers a highly informative and suitable for population genetic studies of moa. Further analyses and interpretations of the genetic diversity are, however, not the scope of this paper and data (including allele frequencies) will be presented elsewhere.

A critical evaluation of the final data confirmed its integrity. One minor deviation from Hardy-Weinberg proportions was observed in the Moa_MA21 locus, but as these data refer to birds living over a span of >4000 years, they do not reflect randomly mating individuals at one point in time. Hardy-Weinberg proportions are not necessarily, therefore, expected by default. Still, the general accordance with expected HW-proportions was an indication that allelic dropout had a minimal effect on the compiled data. Use of additional software, designed specifically to identify scoring problems owing to dropout and stuttering, did not reveal any issues. Lastly, we documented a link between the probability of dropout and C_T_ value, but clearly rejected a negative correlation between observed individual heterozygosity and C_T_ in the compiled data. This also provided strong support for the integrity of the data, indicating that DNA preservation had not affected the final results.

We suggest, therefore, that on the basis of our proposed criteria, the moa microsatellite dataset is of high fidelity despite representing template molecules of c. 600 to 5000 years of age [10]. We argue that variants of the applied methodology will be valid for most scenarios involving aDNA and microsatellites. We emphasise, however, the importance of assessing each case on its merits by including pilot studies and the generation of preliminary data to develop a specific strategy for the material at hand. The proposed Criterion 2 for example, could prove insufficient for types of data where false peaks are difficult to discriminate from true alleles or in situations where contamination is of greater concern.

The achievement of generating a high quality microsatellite dataset for an extinct species does not mean that there is no room for methodological or procedural improvement. A major drawback to the procedure presented here, is the considerable workload associated with single-plexed PCRs, repeated many times with DNA added from one tube at a time. A few experiments with two-plexes were, however, unsuccessful. Methods have been developed for improving microsatellite PCR results from degraded DNA e.g., [19], [25] and further research might elucidate whether our stringent setup can be relaxed somewhat, and whether these novel approaches can be used on aDNA templates without increasing the risk of cross-contamination. However, to generate this first aDNA microsatellite data set, we applied a very simple framework to minimise the number of unknown factors that could compromise the results.

### Concluding remarks

With the six microsatellite markers presented here, the tools are now available for conducting a series of high resolution genetic studies of the extinct New Zealand moa. Although the moa fossils included here are of late Holocene age, and therefore younger than fossils from all the extinct Pleistocene megafauna species, moa were not preserved in very cold environments such as those typical of, for example, woolly mammoth (*Mammuthus primigenieus*), woolly rhino (*Coelodonta antiquitatis*) or steppe bison (*Bison priscus*). With the significant success in amplifying nuclear microsatellite DNA document here, and knowing that low temperatures greatly increases the longevity of aDNA, it seems very likely that our methodology can be applied to research on many other extinct taxa, if they have been preserved in favourable depositional environments.

Like SNPs, microsatellites can offer high resolution genetic insights into the nuclear gene pool of a species. Although widely applied in population genetics on extant species, these two types of markers can be considered novel ‘nuclear alternatives’ in the field of aDNA. In this context though, the markers offer a different set of advantages and disadvantages. For example, microsatellites are often more variable, requiring amplification of fewer loci to achieve a high level of insight, whereas any given SNP can only have a maximum of four states. On the other hand, microsatellites are generally much longer and therefore require better DNA preservation than SNP-based research – a consideration which is most important to LCN aDNA. Issues with allelic dropout will apply to both types of markers although the specific problem of ‘short allele dominance’ is only relevant to microsatellites. In terms of post mortem hydrolytic DNA damage, which is a well-characterised phenomenon in aDNA e.g., [57], it can be difficult to distinguish between a true polymorphism and a damaged site when analysing SNPs. This problem is insignificant in microsatellite-based research, as only allele lengths (and not base composition) are recorded with these markers. So, in short, the decision of which genetic markers to apply has to consider the biomolecule preservation in the substrate at hand, the availability of previously developed markers, as well as the objectives and level of detail the study is aiming at.

We note that generation of ancient microsatellite data and the subsequent verification process presented in this study is only the first step in a series of challenges. Most available methods for analysing microsatellites rely on allele frequencies representing random union of gametes, and this is certainly not so when data are from individuals spanning hundreds of generations. A new viewpoint will be required to take advantage of the information in heterochronous microsatellite data, for example by applying approximate Bayesian computation (ABC) to model and test potential demographic scenarios e.g., [58]. Also, a model allowing analyses of heterochronous microsatellite data is presently being implemented in BEAST [59], which is a widely-applied, coalescent-based, software package for phylogenetic and demographic analysis of sequence data (Alexei Drummond, personal communication).

In this study we have demonstrated how a high fidelity microsatellite data set can be generated from ancient fossils. We emphasise that the results, protocols and considerations presented here are, however, relevant to any type of LCN DNA, regardless of age.

## Supporting Information

Figure S1 **The six GS-FLX reads.** The six original sequences (from GS-FLX data) that yielded functional and polymorphic microsatellite markers in *Dinornis robustus*. Primers are highlighted in red and the repetitive region in yellow.
(DOC)

Figure S2 **Tests for data fidelity.** Tests for genotyping errors using the software MICRO-CHECKER. Deviations from expected homozygote class frequencies proved insignificant and no obvious genotyping errors could be detected in any of the six loci. Results below were copied directly from the output-screen of MICRO-CHECKER.
(DOC)

Table S1 **Seven non-polymorphic markers.** Characteristics of an additional seven microsatellites that amplified well in moa but were excluded from this study because they proved monomorphic or dimorphic in the investigated *Dinornis robustus* samples. Shown here; the moa species in which the microsatellites were identified, repeat units as observed in the original GS-FLX clones, annealing temperatures in the 50-cycle PCR reaction from Allentoft et al. [2], and the primer sequences. * This primerpair was re-designed (shortened) from the generic bird microsatellite TG03-002, presented in Dawson et al. 2009 [43].
(XLS)
