# Supplementary material for: Profiling the Dead: Generating Microsatellite Data from Fossil Bones of Extinct Megafauna—Protocols, Problems, and Prospects
Source: PLoS One. 2011 Jan 31;6(1):e16670. doi: 10.1371/journal.pone.0016670 (PMC3031614; doi:10.1371/journal.pone.0016670)
Supplement: Figure S1 — The six GS-FLX reads. The six original sequences (from GS-FLX data) that yielded functional and polymorphic microsatellite markers in Dinornis robustus. Primers are highlighted in red and the repetitive region in yellow. (DOC) [file pone.0016670.s001.doc]

**Figure S1**

**Moa_MS2:** GenBank #: FJ513189

CCTTACTTTACCCAGCTGGTTAGACAACAGTAATGAGCACCAATACAACTTCATGGTTATGCAATATGCATGGAATATATGTACACACACACACACACACACACACATACATATATAAACAAACATATACTGGAATAGAATAACAGTCTCAAAGAAAA

**Moa_MA1:** GenBank #: HQ823574

CATATAGGCACAAGGAGAGCGCACCCCAATTGGGAAAAAAACACACACACACACACACAGAGTTTACTGGTGACAAGTACACAGATACCATCCTCCCCTGGGCAGCAG

**Moa_MA21:** GenBank #: HQ823575

TGATTGGAGGAGTCTTCGCTTGCAACGTGTCTCGGATGCATAGATACAGGCTTGAAAGCCGGCCAGCTGCTCCGTGTGCGTGTGCGTCTGTGTGTGTGTGTGTGTGTGCAAGGCGCACAGATAACTCCTGCTGC

**Moa_MA38:** GenBank #: HQ823576

AAGAATGCAAGCTGCTTGTTCCCTCCATCACATGCACATACAACATACACACACACACACACAGATGAAGGTGCTAACAGAAAGTGCTGCACAGTGCTGTGAATACAGCCTGTAA

**Moa_MA44:** GenBank #: HQ823577

GCAGACTGGCAAATTTGTGAAATCTGACTCAAACAACCTCCAGCTGATACAGGTTTCTTAGCTTTTCACGACCAAGCTGTGCTTTGGGACTAGGTAACGGTAGGCAAACGCACGGGCTCTGCAGGATTAGATCCCAGGAAGCGGAGAAAGAACAAACAAACAAAGAAACACACACACACACACAGTTTGCAAAGTCCACAGGCTGAGAGCCCAACAACCAGGAA

**Moa_MA46:** GenBank #: HQ823578

GGCCGGTGCGCGGTAACGGTCACCGCCGCCATCGGCTCCCTGAGGGAGGCTGGGGGCTGTCCGCCACTCAAGGGAGCGATTGGGACCGCGGCGAGGAGGAGGAGGAGGAGGAGGAGGAGGAGGACCGAGCCCTTCTC
