# Supplementary material for: Profiling the Dead: Generating Microsatellite Data from Fossil Bones of Extinct Megafauna—Protocols, Problems, and Prospects
Source: PLoS One. 2011 Jan 31;6(1):e16670. doi: 10.1371/journal.pone.0016670 (PMC3031614; doi:10.1371/journal.pone.0016670)
Supplement: Figure S2 — Tests for data fidelity. Tests for genotyping errors using the software MICRO-CHECKER. Deviations from expected homozygote class frequencies proved insignificant and no obvious genotyping errors could be detected in any of the six loci. Results below were copied directly from the output-screen of MICRO-CHECKER. (DOC) [file pone.0016670.s002.doc]

**Figure S2**

**Locus: Moa_MS2**

Probability of observed homozygote class frequency:

Allele Binomial based/ Rank based

118 0.096 0.0695

120 0.9998 1

122 0.2599 0.1595

126 0.9935 1

128 0.9958 1

130 1 1

136 0.2599 0.1525

138 1 1

144 0.0314 0.018

Combined probability for all classes: > 0.05 (not significant)

Total expected homozygotes: 14.847

Total observed homozygotes: 16

No evidence for scoring error due to stuttering.

No evidence for large allele dropout.

No evidence for null alleles.

Locus: **Moa_MA1**

Probability of observed homozygote class frequency:

Allele Binomial based/ Rank based

91 0.6076 0.4945

92 0.9969 1

93 0.996 1

95 0.4868 0.4085

97 0.7959 0.622

99 0.9988 1

Combined probability for all classes: > 0.05 (not significant)

Total expected homozygotes: 21.081

Total observed homozygotes: 20

No evidence for scoring error due to stuttering.

No evidence for large allele dropout.

No evidence for null alleles.

**Locus: Moa_MA21**

Probability of observed homozygote class frequency:

Allele Binomial based/ Rank based

93 0.9988 1

97 0.0998 0.068

107 0.2376 0.1975

109 0.0133 0.009

111 0.9996 1

113 0.3656 0.2985

Combined probability for all classes: > 0.05 (not significant)

Total expected homozygotes: 22.358

Total observed homozygotes: 28

No evidence for scoring error due to stuttering.

No evidence for large allele dropout.

No evidence for null alleles.

**Locus: Moa_MA38**

Probability of observed homozygote class frequency:

Allele Binomial based/ Rank based

84 0.0577 0.031

86 0.164 0.0985

90 0.4938 0.434

92 0.1041 0.067

96 0.9151 0.8015

98 0.9996 1

100 0.9992 1

102 0.9998 1

104 1 1

110 0.4223 0.26

132 1 1

Combined probability for all classes: > 0.05 (not significant)

Total expected homozygotes: 11.729

Total observed homozygotes: 15

No evidence for scoring error due to stuttering.

No evidence for large allele dropout.

No evidence for null alleles.

**Locus: Moa_MA44**

Probability of observed homozygote class frequency:

Allele Binomial based/ Rank based

75 0.0541 0.035

79 0.4418 0.415

83 0.1381 0.0985

85 0.6333 0.491

Combined probability for all classes: > 0.05 (not significant)

Total expected homozygotes: 26.554

Total observed homozygotes: 30

No evidence for scoring error due to stuttering.

No evidence for large allele dropout.

No evidence for null alleles.

**Locus: Moa_MA46**

More than 50% of the alleles at this locus are of one allele size class.

Binomial analysis could not be performed.

Total expected homozygotes: 28.864

Total observed homozygotes: 29

No evidence for scoring error due to stuttering.

No evidence for large allele dropout.

No evidence for null alleles.
